# Seasonality of medically attended norovirus gastroenteritis and its association with climatic factors within an US integrated healthcare system, 2016–2019

**DOI:** 10.1371/journal.pone.0318077

**Published:** 2025-05-09

**Authors:** Claire P. Mattison, Laura E. Calderwood, Jordan E. Cates, Judy Donald, Aron J. Hall, Mark A. Schmidt, Sara A. Mirza

**Affiliations:** 1 Centers for Disease Control and Prevention, Atlanta, Georgia, United States of America; 2 Cherokee Nation Operational Solutions, Tulsa, Oklahoma, United States of America; 3 Kaiser Permanente Northwest, Portland, Oklahoma, United States of America; National Center for Global Health and Medicine, JAPAN

## Abstract

**Background:**

While acute gastroenteritis (AGE) occurs year-round, norovirus has a winter seasonality in the United States.

**Objective:**

We analyzed norovirus seasonality within a US integrated healthcare delivery system from 2016–2019.

**Methods:**

Electronic medical records were collected for acute gastroenteritis (AGE) encounters with specific ICD-9/10 codes or clinical stool testing. Norovirus percent positivity was calculated as the 8-week centered rolling average. Temperature and absolute humidity data were measured via weather station. The relationship between these factors and weekly norovirus episodes were modeled via negative binomial models.

**Results:**

From 2016–2019, there were 198,181 AGE episodes reported; among the 18,998 episodes tested, 892 (5%) were norovirus positive. Norovirus percent positivity peaked in epidemiologic week 7 at 9%. Two negative binomial models showed significant inverse relationships between weekly number of norovirus episodes and both temperature and absolute humidity.

**Conclusion:**

Norovirus AGE exhibited winter seasonality from 2016–2019, associated with lower temperatures and humidity. Understanding this seasonality may help predict peak transmission periods and their impact on healthcare resources.

## Introduction

Viral acute gastroenteritis (AGE), defined as diarrhea or vomiting caused by viruses such as rotavirus and norovirus, has a substantial global disease burden [1]. While rotavirus is the leading cause of AGE among children <5 years old worldwide [2], in countries that have introduced rotavirus vaccine, such as the United States, norovirus is now the leading cause of AGE [3].

While viruses cause AGE year-round, viral AGE tends to have a winter seasonality [4–5]. Norovirus has been defined by its winter seasonality and was originally known as “winter vomiting disease” [6]. US-based surveillance systems of AGE outbreaks and sporadic disease also show a winter seasonality for norovirus, starting in November and peaking in December/January [7,8]. The US rotavirus season typically begins in January and peaks mid-spring; post-vaccine introduction, rotavirus has developed a biennial pattern with alternating high and low seasons [9].

The exact factors that influence seasonality of viral AGE are unknown but may include human behaviors, virologic factors, and climatic factors, such as temperature, humidity, and rainfall. A review of studies looking at the relationship of various climatic factors and norovirus outbreaks found lower temperatures were correlated with periods of high norovirus transmission, while the relationship between norovirus and humidity was mixed [10]. Studies reviewed found both positive and negative correlations between relative humidity and norovirus transmission [10–12]. The relationship between rainfall and norovirus was also unclear; some studies found that norovirus was correlated with rainfall while others found a correlation with dry weather [10]. While the exact mechanism of how climate factors such as; temperature, humidity, and rainfall may interact to impact norovirus stability and transmission is unknown, it is possible these factors may both change human behavior and influence virus stability and allow for increased transmission. Additionally, temperature extremes may change human behavior, increasing time spent indoors in possibly crowded conditions resulting in increased contacts, and conditions for increased disease transmission. Understanding the relationship between climatic factors and AGE may help predict periods of increased disease transmission and future changes to disease transmission dynamics in the face of continued climate change.

While a winter seasonality for viral AGE pathogens is seen in other large US surveillance systems, few studies have had the ability to analyze the healthcare burden and seasonality of sporadic norovirus AGE across all ages. The goal of this analysis is to better understand the seasonal burden of all-cause and norovirus-associated AGE on the healthcare system and its relationship to climatic factors using electronic medical record data from the Kaiser Permanente Northwest (KPNW) healthcare system. Since KPNW data was collected from a distinct geographic area, we had the opportunity to dive more deeply into the data to understand what factors could be impacting the seasonality of viral AGE. We analyzed the seasonality of medically attended AGE in across all age groups and medical settings and modeled the relationship between norovirus-associated AGE and both temperature and absolute humidity.

## Methods

### Study site

Data were collected from the Kaiser Permanente Northwest (KPNW) healthcare system, which serves approximately 600,000 people centered in the Portland, Oregon metropolitan area, including areas of southern Washington state and northern and central Oregon [13]. Portland is in the Willamette Valley with a temperate seasonality and average monthly temperatures ranging between 40–70°F (4–21°C) throughout the year; extreme temperatures range from the single digits in December and January to over 100°F (38°C) in June, July, and August [14].

### Dataset criteria

The dataset included all healthcare encounters for acute gastroenteritis (AGE) from January 1, 2016 to December 31, 2019. Data were accessed from the electronic health record for research purposes on September 7^th^, 2020. AGE encounters were identified via ICD-9/10 code (S1 Table) or a clinician ordered stool laboratory test (e.g., stool culture, *Clostridium difficile* test, parasitic test, or PCR panel) (S2 Table). A medically attended AGE episode was defined as all AGE encounters occurring within 30 days of each other. Episodes were classified based on the highest level of care received across included encounters; level of care was ranked as inpatient, emergency department, urgent care, outpatient, or remote (email or telephone) from highest to lowest, respectively. A single patient could have multiple AGE episodes during the study period. Data on clinical stool testing, as well as demographics, such as age, sex, and race, were collected from electronic health records. Age (in years) was defined at episode start date.

To reduce the likelihood of including chronic AGE patients, episodes >30 days were excluded. Additionally, patients classified as having a chronic AGE ICD-9/10 code and no ICD-9/10 code for vomiting at their encounter were excluded (S3 Table). Dialysis encounters were also excluded, regardless of ICD-9/10 codes or laboratory orders linked to those encounters.

### Data classification

A clinical test was linked to an AGE episode if ordered or completed within 7 days of the start or end of an episode. On January 20, 2016, KPNW introduced PCR panel viral stool testing via the xTAG® Gastrointestinal Pathogen Panel (Luminex Molecular Diagnostics, Austin, TX, USA) and on September 23, 2019, switched to enteric bacterial, parasitic, and viral BD MAX^TM^ PCR panels (BD Diagnostics, Baltimore, MD, USA). An AGE episode was classified as norovirus or rotavirus positive if a linked PCR panel test was positive for either pathogen.

AGE episodes were categorized into 52 epidemiological weeks (EW) per year by episode start date. All EW were defined to begin on a Sunday and EW 1 was assigned as the first week with at least four days within that calendar year [15]. Norovirus surveillance year was defined as EW 27 to EW 26 of the following calendar year [13]. Rotavirus surveillance year was defined by calendar year. Seasonal onset was defined as the EW where 10% of the cumulative seasonal cases for norovirus or rotavirus had occurred, and offset was defined as the EW where 90% of the cumulative seasonal cases had occurred [7]. Season length was defined as the number of EW between onset and offset. Weekly percent positivity was calculated as the number of norovirus or rotavirus associated episodes divided by the number of episodes with testing as an 8-week centered rolling average.

### Climate data

Daily weather data during the study period were obtained from the Portland International Airport weather station. Temperature data were accessed via the Global Historical Climatology Network [16]. Relative humidity data were accessed from the Integrated Surface Dataset [17]. Both datasets were compiled by the National Centers for Environmental Information, National Oceanic and Atmospheric Administration. Since relative humidity is a measure of the amount of water vapor in the air compared to the maximum amount possible and changes with air temperature, we converted relative humidity to absolute humidity to assess temperature and humidity separately. Absolute humidity (g/m^3^) was calculated using the following equation with maximum daily temperature (T) and maximum daily relative humidity (rh): $Absolute\;Humidity\;(\frac{grams}{m^{3}})=\;\frac{6.112\;\times \;e^{\left[\frac{17.67\times T}{T+243.5}\right]\;}\times \;rh\;\times \;2.1674}{273.15\;+T}$ [18]. Weekly climate measures were calculated by averaging daily values over the EW.

### Statistics

Two negative binomial models were used to assess the relationship between the weekly number of norovirus positive episodes and climatic factors utilizing data from mid-2016 (EW 27) through the end of 2019. The first model used maximum temperature as a predictor and second used absolute humidity. A time lag between climate measurements and number of positive episodes from 0 to 4 weeks was analyzed. Interaction terms with both age (<18 years, > 18 years) and level of care (outpatient, more severe than outpatient) were also investigated. Model fit was determined by the Akaike information criterion (AIC). Due to the small number of rotavirus positive episodes in our sample (n = 204), the relationship between weekly rotavirus positive episodes and climatic factors could not be analyzed.

### Ethics

This project was reviewed and approved by the Kaiser Permanente Northwest Institutional Review Board (FWA00002344). The institutional review board granted this study a waiver of informed consent for this data, as all data was collected from the electronic medical record. Our dataset did not include individuals who have opted their medical records out of Kaiser Permanente Research activities.

## Results

From 2016 to 2019, 132,156 persons presented to Kaiser Permanente Northwest for 198,181 medically attended AGE episodes (Table 1). The majority (72%) of individuals with a medically attended AGE episode experienced only one episode during our study period. Annually, the number of AGE episodes ranged from 44,532 episodes in 2016–53,399 episodes in 2019, increasing over the 4-year period.

**Table 1:** Demographic characteristics of medically attended acute gastroenteritis episodes.

	AGE Episodes N (%)	AGE Episodes with PCR panel testing N (%)	Norovirus positive^c^ AGE Episodes N (%)	Rotavirus positive^c^ AGE episodes N (%)
Total number of episodes^a^	198,181	18,998	892	204
Age				
<5 years old	11,244 (6)	1,090 (6)	150 (17)	47 (23)
5-17 years old	11,762 (6)	1,267 (7)	66 (7)	34 (17)
18-64 years old	111,588 (56)	11,415 (60)	519 (58)	92 (45)
65 + years old	63,587 (32)	5,226 (28)	157 (18)	31 (15)
Sex				
% Female	115,369 (58)	11,424 (60)	441 (49)	104 (51)
Race				
White	154,785 (78)	15,523 (82)	644 (72)	148 (73)
Black	7,469 (4)	491 (3)	28 (3)	4 (2)
Asian	8,456 (4)	639 (3)	39 (4)	11 (5)
Another race or multiracial	9,340 (5)	834 (4)	48 (5)	9 (4)
Unknown race	18,302 (9)	1,511 (8)	133 (15)	32 (16)
Highest level of care^b^				
Remote	14,191 (7)	123 (1)	2 (0)	0 (0)
Outpatient	87,960 (44)	13,520 (71)	462 (52)	105 (51)
Urgent care	14,680 (7)	2,621 (14)	212 (24)	49 (24)
Emergency Department	32,518 (16)	1,490 (8)	159 (18)	40 (20)
Inpatient	48,832 (25)	1,244 (7)	57 (6)	10 (5)

Results stratified by enteric PCR panel testing and viral positivity in Kaiser Permanente Northwest, Portland, Oregon, 2016–2019

^a^AGE encounters were identified via AGE ICD 9/10 code or infectious stool laboratory test. All AGE encounters occurring within 30 days of each other were consolidated into a single AGE episode. Episodes longer than 30 days were excluded. A single patient may be represented by multiple episodes within the dataset.

^b^Encounters were classified into remote, outpatient, urgent care, emergency department, or inpatient based on encounter type. Episodes (encounters within a 30-day period) were classified by highest level of care, with inpatient as highest level and remote as lowest, respectively.

^c^At least one positive PCR panel test for the pathogen during an AGE episode

Almost half (44%) of episodes were classified as outpatient, while 7% were urgent care, 16% were emergency department, and 25% were inpatient (Table 1). Seven percent of episodes were classified as remote. The median age of an AGE patient was 52 years old (range: 0–106 years); 6% were under 5 years old, and a third were 65 years or older (Table 1). There was no difference in the number of AGE episodes that occurred from October to March (51%) versus from April to September (49%).

**Fig 1:**
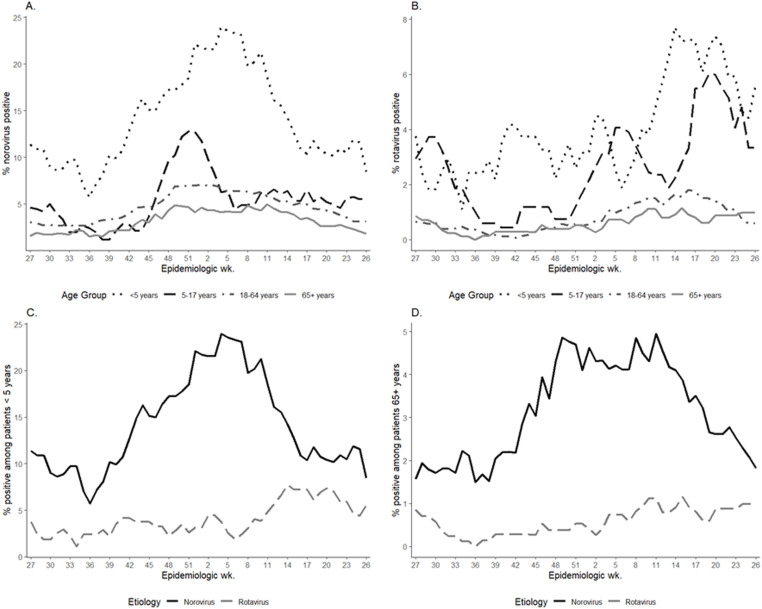
Percent positivity of norovirus and rotavirus by epidemiologic week and age group. Eight week rolling average of viral AGE percent positivity by epidemiologic week of episode*, Kaiser Permanente Northwest, Portland, OR, USA, 2016–2019; A) Norovirus positivity among all age groups, B) Rotavirus positivity among all age groups, C) Norovirus and rotavirus positivity among patients <5 years old, D) Norovirus and rotavirus positivity among patients 65 + years old. *All AGE encounters occurring within 30 days of each other were consolidated into a single AGE episode. A single patient may be represented by multiple episodes within the dataset.

Of all AGE episodes, 10% (n = 18,998) had stool tested for AGE pathogens via PCR panel. This varied by setting; 18% of urgent care episodes had PCR panel testing, followed by 15% of outpatient episodes, 5% of emergency department episodes, and 3% of inpatient episodes (Table 1). Less than 1% of remote episodes had testing. Of episodes with testing, 12% were positive for at least one pathogen; 892 (5%) were norovirus positive and 204 (1%) were rotavirus positive. Patients under 5 years old had the highest percent positivity for norovirus (14%) and rotavirus (4%). Percent positivity decreased with each age group for norovirus and rotavirus; 5–17 year-olds had 5% and 3% of healthcare episodes positive while, 18–64 year-olds had 4% and 1% of episodes positive, respectively. Those over 65 years old had the lowest percent positivity (3% for norovirus; 1% for rotavirus) throughout the year (Fig 1). Percent positivity for *any* pathogen ranged from 10.5% in October to 15.3% in December. Other commonly detected pathogens included *Campylobacter* (3%), *Salmonella* (1%) and *E. coli* (1%).

**Fig 2:**
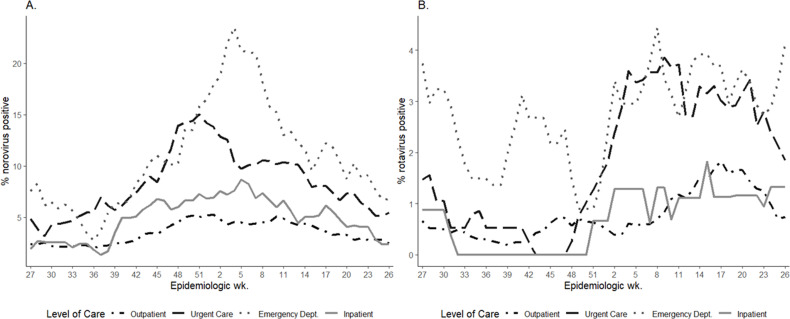
Percent positivity of norovirus and rotavirus by epidemiologic week and level of care. Eight week rolling average of viral AGE percent positivity by epidemiologic week of episode* and highest level of care sought^†^, Kaiser Permanente Northwest, Portland, OR, USA, 2016–2019. A) Norovirus positivity among all levels of care, B) Rotavirus positivity among all levels of care. *All AGE encounters occurring within 30 days of each other were consolidated into a single AGE episode. A single patient may be represented by multiple episodes within the dataset. ^†^Encounters were classified into outpatient, urgent care, emergency department, or inpatient based on encounter type. Episodes were classified by highest level of care, with inpatient as highest level and outpatient and lowest.

From mid-2016 (EW 27) to mid-2019 (EW 26), norovirus percent positivity on average peaked in EW 7 (mid-February) at 9% and was lowest in EW 34 (late-August) at 2%. On average, norovirus seasonal onset occurred in EW 34 (late-August) and the offset was by EW 19 (early-May). Across all surveillance years combined, norovirus seasonal peaks were higher for inpatient (9%), emergency department (23%), and urgent care (15%) episodes compared to outpatient episodes (5%) (Fig 2).

**Fig 3:**
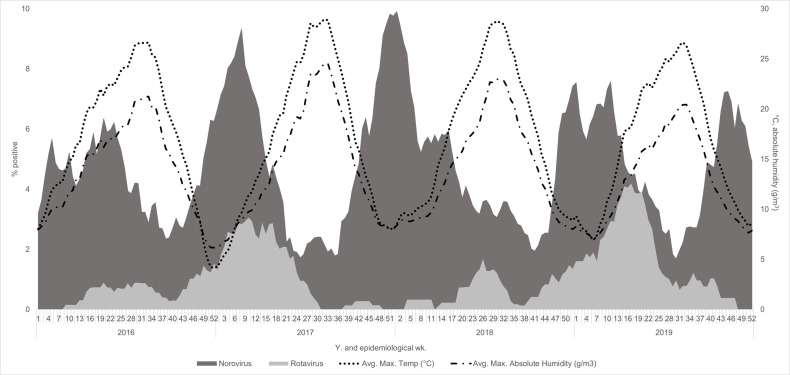
Norovirus and rotavirus percent positivity and climate factors. Eight week rolling average of viral AGE percent positivity, plotted with temperature and absolute humidity, Kaiser Permanente Northwest, Portland, OR, USA, 2016–2019.

Rotavirus percent positivity had different seasonal patterns in odd and even years. In 2017 and 2019, the rotavirus season had an onset in EW 5–6 and offset between EW 25–34 and peaked in EW 14 with a percent positivity of 4%. In 2016 and 2018, rotavirus had a later and less distinct season, with onset in EW 18–19 and offset in EW 49 and peaked in EW 39 at 2% (Fig 3).

Temperature and absolute humidity peaked in the summer and were the lowest in the winter, displaying an opposite seasonal pattern to norovirus positivity (Fig 3). Using data from mid-2016 (EW 27) through the end of 2019 in a negative binomial model, we found temperature had a significant inverse relationship with the weekly count of norovirus positive AGE episodes (Table 2). A three-week time lag between temperature and norovirus positive episodes had the best model fit. For every 5-degree Celsius increase in the maximum weekly temperature, three weeks later weekly counts of norovirus positive AGE episodes decreased by 21% (95% CI: 16–25%). Absolute humidity also had a significant inverse relationship with the weekly count of norovirus positive AGE episodes (Table 3). For this model, a two-week time lag had the best model fit. For every 5 g/m^3^ increase in absolute humidity, two weeks later, weekly counts of norovirus positive AGE episodes decreased by 28% (95% CI: 22–33%) (Table 3). We found no significant interactions between these relationships by age group (<18 years old vs. > 18 years old) or encounter type (outpatient vs. more severe than outpatient).

**Table 2:** Maximum temperature (°C) as a predictor of the weekly count of norovirus positive AGE episodes.

Predictor: Maximum Temperature (°C)					
Lag^*^ (weeks)	Model Coefficient	p-value	AIC^†^	Relative Risk (95% CI) per 1 unit increase	Percent decrease (95% CI) per 5-unit increase
0	‒0.0391	<0.0001	831.9	0.962 (0.951-0.973)	17.8% (12.9%-22.3%)
1	‒0.0412	<0.0001	826.0	0.960 (0.949-0.970)	18.6% (14.0%-23.0%)
2	‒0.0458	<0.0001	813.6	0.955 (0.945-0.966)	20.5% (16.0%-24.7%)
3	‒**0.0466**	**<0.0001**	**810.9**	**0.954 (0.944-0.965)**	**20.8% (16.4%-24.9%)**
4	‒0.0459	<0.0001	813.1	0.955 (0.945-0.966)	20.5% (16.1%-24.6%)

Negative binomial model results on maximum temperature (°C) as a predictor of the weekly count of norovirus positive AGE episodes, Kaiser Permanente Northwest, Portland, OR, USA, July 2016–December 2019

* Time lag in weeks between measurement of climate factors and number of norovirus episodes

† Akaike Information Criterion (AIC) as an estimate of prediction error where a lower value indicates a better fit.

**Table 3:** Absolute humidity (g/m^3^) as a predictor of the weekly count of norovirus positive AGE episodes.

Predictor: Absolute Humidity (g/m^3^)					
Lag^*^ (weeks)	Model Coefficient	p-value	AIC^†^	Relative Risk (95% CI) per 1 unit increase	Percent decrease (95% CI) per 5-unit increase
0	-0.054	<0.0001	832.9	0.947 (0.932-0.963)	23.7% (17.3%-29.6%)
1	-0.057	<0.0001	828.6	0.945 (0.930-0.960)	24.6% (18.4%-30.4%)
**2**	**-0.064**	**<0.0001**	**814.9**	**0.938 (0.923-0.952)**	**27.5% (21.7%-33.0%)**
3	-0.064	<0.0001	815.8	0.938 (0.923-0.953)	27.4% (21.5%-32.9%)
4	-0.064	<0.0001	816.8	0.938 (0.924-0.953)	27.2% (21.3%-32.7%)

Negative binomial model results on absolute humidity (g/m3) as a predictor of the weekly count of norovirus positive AGE episodes, Kaiser Permanente Northwest, Portland, OR, USA, July 2016–December 2019

* Time lag in weeks between measurement of climate factors and number of norovirus episodes

† Akaike Information Criterion (AIC) as an estimate of prediction error where a lower value indicates a better fit.

## Discussion

In this integrated healthcare system in Portland, Oregon, AGE was associated with nearly half a million medically-attended AGE encounters during 2016‒2019. Across all healthcare settings and ages, norovirus-associated AGE represented a significant burden and displayed a winter seasonality, peaking in January and February. Norovirus-associated AGE seasonal peaks were the highest in younger age groups, urgent cares, and emergency departments, demonstrating the increased burden norovirus-AGE has among these age groups and in these healthcare settings. Rotavirus-associated AGE showed a biennial seasonality and spring peak. Using two negative binomial models, both temperature and absolute humidity were inversely correlated with the weekly number of norovirus-positive healthcare episodes.

These results align with previous reports of the significant burden [5,19] and seasonal nature of norovirus and rotavirus in the US [7,20]. Consistent with patterns shown since US rotavirus vaccine introduction, rotavirus associated AGE displayed a biennial pattern, with more during odd years, and less during even years [9]. While individual rotavirus vaccine data was not available for this population, other studies have shown >75% coverage among age-eligible children within Kaiser Permanente Northwest [21]. Since rotavirus vaccine introduction, vaccine coverage and birth rate have been found to have an impact on US rotavirus seasonality, independent of climatic factors [22]. In alignment with other surveillance systems and publications on sporadic norovirus illness and outbreaks, norovirus had a winter seasonality in this system. [7 20]. We saw the highest norovirus percent positivity among those <5 years old, and norovirus percent positivity decreased with age. This finding aligns with the higher burden of norovirus among children <5 years shown in other large-scale datasets [19]. With norovirus vaccine candidates under development [23], norovirus seasonal patterns may also change after vaccine introduction, depending on the vaccine’s target population and uptake.

Data from other countries have found a relationship between temperature and norovirus [12,24,25]. Predictive models from South Korea and China found that cold temperature was the most important climatic predictor of norovirus outbreaks [26,27]. A Hong Kong based study found low temperature and humidity extremes were the best predictors of pediatric AGE hospital admissions [28] Experimental studies have found that norovirus persisted in water and on surfaces longer at lower temperatures, showing that virus survivability and transmissibility may be longer during in the winter season [29,30]. In agreement, our study found that as temperature decreased, norovirus-associated AGE increased; our model had the best fit with a three-week time lag, meaning that, temperature best predicted norovirus-associated AGE episodes three weeks later.

In this study, we analyzed the relationship between norovirus-associated AGE and absolute, as opposed to relative, humidity. Absolute humidity measures the amount of water vapor (moisture) in the air, regardless of temperature, whereas relative humidity is the percentage of the amount of water vapor present compared to amount needed for saturation at the air’s temperature. For this reason, to separate potential impacts on norovirus from temperature and humidity, we focused on absolute and not relative humidity. Indoor and outdoor absolute humidity are also highly correlated; therefore, fluctuations in outdoor absolute humidity may impact norovirus transmission in indoor settings where outbreaks commonly occur [31]. Laboratory studies have found that absolute humidity has a large impact on norovirus survivability and infectiousness and low absolute humidity improves the survival of human norovirus surrogates [32]. A Swedish study found that a drop in absolute humidity coincided with an increase in norovirus outbreaks [33]. This is consistent with our study findings, where the highest number of norovirus-associated healthcare episodes occurred when absolute humidity was the lowest. Other studies have found relationships between AGE and rainfall, hydrology, and land development use [10,11]. However, we did not analyze these factors in this study.

This study was unique in being able to analyze the impact of climatic factors on norovirus-associated AGE episodes, something few US based studies have been able to do. However, this study is subject to multiple limitations. The use of electronic healthcare record data allowed us to utilize four years of data and hundreds of thousands of medical encounters and clinical tests along with corresponding climatic data for the same years. However, data were selected based on ICD-9/10 codes and clinical testing and most patients did not have stool tests performed, limiting the data available and potentially introducing bias. Additionally, we know from other Kaiser Permanente studies that AGE patients seeking healthcare are more likely to have more severe disease and a longer duration of symptoms than those who not seeking healthcare [34]. Additionally, as an ecological study, we do not have data to analyze exposures or biases at the individual level. Our negative binomial models assume a linear relationship, which may not reflect the complexity of the interaction between climatic factors and disease. While we found a significant relationship for norovirus-associated episodes, sample sizes restricted our ability to run models for rotavirus-associated episodes or control for any confounders. We examined different interactions between age and encounter type, which were not significant. These models also do not give us the ability to assess causality, only correlation. With absolute humidity and temperature highly correlated with each other, it is unclear which factor may be influencing norovirus transmission. As well, it is not possible to tell how these factors are directly impacting viral survivability and transmissibility or are influencing may behavior and subsequently transmission.

Behavior, regardless of climatic factors, can have strong impact on AGE transmission. This was seen during the COVID-19 pandemic; AGE incidence and outbreaks decreased substantially when non-pharmaceutical interventions were put into place in 2020 and 2021, such as social distancing and enhanced cleaning measures [35–37]. However, we did not have data on seasonal behavior changes that may impact norovirus disease dynamics. The US school year typically begins in August or September and multiple holidays, including Thanksgiving (in late November), Christmas (in late December) and New Years (on January 1^st^), align with the winter season and provide opportunities for large gatherings to drive disease transmission. In Belgium, an influenza transmission study found that person-to-person contacts increased significantly on workdays compared to weekends and holidays, while temperature, rainfall, and humidity had a minor influence on the number of contacts [38]. Other studies have found that norovirus season in children often precedes that among older age groups, potentially indicating transmission in settings such as schools and childcares drives norovirus seasonality [39]. However, in this study, children had higher and sharper seasonal peaks but did not peak earlier than older age groups.

Climate change is expected to influence infectious diseases patterns globally [40]. Better understanding the impact of climate on AGE seasonality is key to preparing prevention efforts and predicting future shifts in AGE burden and seasonality as the climate warms. This study provides data on the burden and seasonality of AGE in a healthcare system to improve understanding of medically-attended AGE and inform timing of prevention efforts, including promotion of proper disinfection techniques, hand hygiene, and isolation of ill individuals, as well as future vaccination campaigns. Understanding how AGE burden varies throughout the year can help healthcare systems plan resources and prepare for times of peak transmission. Moving forward, more studies looking at the relationship between climatic factors, particularly temperature and absolute humidity and norovirus are warranted.

## Supporting information

S1 Table International Classification of Disease – Clinical Modification version 9 (ICD-9) and version 10 (ICD-10) codes used to identify healthcare encounters associated with acute gastroenteritis, Kaiser Permanente Northwest, Portland, Oregon, USA, 2016–2019. (DOCX)

S2 Table Clinical stool tests used to identify healthcare encounters associated with acute gastroenteritis, Kaiser Permanente Northwest, Portland, Oregon, USA, 2016–2019. (DOCX)

S3 Table International Classification of Disease – Clinical Modification version 9 (ICD-9) and version 10 (ICD-10) codes used to identify patients with chronic acute gastroenteritis, Kaiser Permanente Northwest, Portland, Oregon, USA, 2016–2019. (DOCX)
