# Supplementary material for: Seasonality of medically attended norovirus gastroenteritis and its association with climatic factors within an US integrated healthcare system, 2016–2019
Source: PLoS One. 2025 May 9;20(5):e0318077. doi: 10.1371/journal.pone.0318077 (PMC12063862; doi:10.1371/journal.pone.0318077)
Supplement: S1 Table — (DOCX) [file pone.0318077.s001.docx]

**S1 Table.** International Classification of Disease – Clinical Modification version 9 (ICD-9) and version 10 (ICD-10) codes used to identify healthcare encounters associated with acute gastroenteritis, Kaiser Permanente Northwest, Portland, Oregon, USA, 2016–2019

| **Diagnosis** | **ICD-9** | **ICD-10** |
| --- | --- | --- |
| Cause unspecified |  |  |
| Presumed infectious | 009.0-009.3 | A09 |
| Presumed noninfectious | 558.9 | K52.9 |
| Symptom: Diarrhea NOS | 787.91 |  |
| Nausea and vomiting | 787.0 | R11 |
| Nausea with vomiting | 787.01 | R11.2 |
| Vomiting, alone (NOS) | 787.03 | R11.10 |
| Projectile vomiting |  | R11.12 |
| Cause specified |  |  |
| Viral | 008.61-008.8 | A08.0-A08.5 |
| Rotavirus | 008.61 | A08.0 |
| Adenovirus | 008.62 | A08.2 |
| Norwalk | 008.63 | A08.1 |
| Other viral enteritis | 008.64-008.69 | A08.3, A08.5 |
| Other not elsewhere classified | 008.8 | A08.4 |
| Bacterial | 001.0-005.9, 008.0-008.5 | A00.0-A05.9 |
| Cholera | 001.0-00.9 | A00.0-A00.9 |
| Typhoid/Paratyphoid | 002.0-002.9 | A01.0-A01.4 |
| Salmonella | 003.0-003.9 | A02.0-A02.9 |
| Shigella | 004.0-004.9 | A03.0-A03.9 |
| Other bacterial food poisoning | 005.0-005.9 | A05.0-A05.9 |
| E. coli | 008.0 | A04.0-A04.4 |
| Other/unspecified bacteria | 008.1-008.5 | A04.5-A04.9 |
| Parasitic | 006.0-006.2, 006.9-007.9 | A06.0-A07.9 |
| Ameba | 006.0-006.2, 006.9 | A06.0-A06.2 |
| Other protozoal | 007.0-007.9 | A07.0-A07.9 |
