# Supplementary material for: Seasonality of medically attended norovirus gastroenteritis and its association with climatic factors within an US integrated healthcare system, 2016–2019
Source: PLoS One. 2025 May 9;20(5):e0318077. doi: 10.1371/journal.pone.0318077 (PMC12063862; doi:10.1371/journal.pone.0318077)
Supplement: S2 Table — (DOCX) [file pone.0318077.s002.docx]

**S2 Table.** Clinical stool tests used to identify healthcare encounters associated with acute gastroenteritis, Kaiser Permanente Northwest, Portland, Oregon, USA, 2016–2019

| Type of Test | Test name (from Kaiser Permanente Northwest database) |
| --- | --- |
| Antigen | Clostridioides (clostridium) difficile antigen and toxins a and b with reflex to PCR |
| Antigen | Cryptosporidium antigen, EIA |
| Antigen | Cryptosporidium, stool, DFA |
| Antigen | Entamoeba histolytica antigen |
| Antigen | Giardia lamblia antigen, EIA |
| Antigen | Helicobacter pylori antigen, stool |
| Antigen | Rotavirus antigen |
| Culture | Stool culture (salmonella, shigella, campylobacter, e. Coli 0157) |
| Culture | Vibrio culture, stool |
| Culture | Yersinia species culture |
| PCR | Clostridioides (clostridium) difficile, PCR |
| PCR | GI virus panel (adenovirus, astrovirus, norovirus, rotavirus, sapovirus), stool, multiplex PCR |
| PCR | Stool, multiplex PCR |
| Smear | Cyclospora and cystoisospora examination, stool, acid-fast stain |
| Smear | Cyclospora smear, acid fast stain |
| Smear | Isospora smear |
| Smear | Microsporidia smear, stool |
| Smear | Ova and parasites examination, concentration, direct smear and special stain |
| Smear | Parasite exam, stool, concentration with direct smear and stain |
