# Supplementary material for: Seasonality of medically attended norovirus gastroenteritis and its association with climatic factors within an US integrated healthcare system, 2016–2019
Source: PLoS One. 2025 May 9;20(5):e0318077. doi: 10.1371/journal.pone.0318077 (PMC12063862; doi:10.1371/journal.pone.0318077)
Supplement: S3 Table — (DOCX) [file pone.0318077.s003.docx]

**S3 Table.** International Classification of Disease – Clinical Modification version 9 (ICD-9) and version 10 (ICD-10) codes used to identify patients with chronic acute gastroenteritis, Kaiser Permanente Northwest, Portland, Oregon, USA, 2016–2019

| **Diagnosis** | **ICD-9** | **ICD-10** |
| --- | --- | --- |
| Whipple’s disease | 40.2 | K90.81 |
| Malignant neoplasm of any part of esophagus | 150 | C15 |
| Malignant neoplasm of any part of stomach | 151 | C16 |
| Malignant neoplasm of any part of small intestine | 152 | C17 |
| Malignant neoplasm of any part of large intestine | 153 |  |
| Malignant neoplasm of colon |  | C18 |
| Malignant neoplasm of any part of rectum rectosigmoid junction and anus | 154 | C19 |
| Malignant neoplasm of rectum |  | C20 |
| Malignant neoplasm of anus and anal canal |  | C21 |
| Malignant neoplasm of liver | 155 | C22 |
| Malignant neoplasm of gallbladder | 156 | C23 |
| Malignant neoplasm of extrahepatic bile duct |  | C24 |
| Malignant neoplasm of pancreas | 157 | C25 |
| Malignant neoplasm of peritoneum | 158 | C48 |
| Malignant neoplasm of intestinal tract part unspecified, spleen, other sites of digestive system and intra-abdominal organs, neoplasm of ill-defined sites, etc. | 159 | C26 |
| Crohn's disease [regional enteritis] | 555 | K50 |
| Ulcerative chronic colitis | 556 | K51 |
| Irritable bowel syndrome | 564.1 | K58 |
| Celiac disease | 579 | K90.0 |
| Personal history of malignant neoplasm of stomach | V10.04 | Z85.028 |
| Personal history of malignant neoplasm of large intestine | V10.05 | Z85.038 |
